# Children’s Experience of Posttraumatic Growth: Distinguishing General from Domain-Specific Correlates

**DOI:** 10.1371/journal.pone.0145736

**Published:** 2015-12-29

**Authors:** Odilia M. Laceulle, Rolf J. Kleber, Eva Alisic

**Affiliations:** 1 Medical and Clinical Psychology, Tilburg University, Tilburg, the Netherlands; 2 Clinical & Health Psychology, Utrecht University, Utrecht, the Netherlands; 3 The Netherlands & Foundation Arq, Diemen, the Netherlands; 4 Monash Injury Research Insititute, Monash University, Australia & National Psychotrauma Center for Children and Youth, UMC Utrecht, Utrecht, the Netherlands; University of Stellenbosch, SOUTH AFRICA

## Abstract

Although the five domains of posttraumatic growth (new possibilities, relating to others, personal strength, spiritual change and appreciation of life) have been studied extensively in adults, little is known about these domains and their correlates in children. We aimed to examine whether demographic and/or social characteristics are related to children’s reports of overall posttraumatic growth and of growth in specific domains. In a general population study, children aged 8–12 years who had been exposed to adverse events (*N* = 1290) filled out questionnaires on their experiences, demographic characteristics (gender, age, time lag since event), stress reactions, peer support, religiosity and posttraumatic growth. All demographic and social characteristics were related to overall posttraumatic growth, except time lag. Associations varied across the five domains with the strongest effects being found for stress reactions and religiosity. A higher level of stress reactions was related to more growth in all domains (general effect), whereas religious children experienced more spiritual growth than non-religious children without differences on other domains (domain specific effect). Other effects were small, and some did not remain significant after Bonferroni corrections. These findings suggest the presence of both general and domain-specific correlates of child posttraumatic growth. Although effects were generally small, the current findings show the need to differentiate between the domains of posttraumatic growth in both further research and clinical practice. This will allow a better understanding of the mechanisms of posttraumatic growth in children as well as more tailored assessment and intervention.

## Introduction

Traditionally, research on stress and trauma has focused on negative sequelae of adversity. Over the last decade an increasing emphasis has been placed on growth in the aftermath of adversity. Posttraumatic growth, described as reflecting “positive change experienced as a result of the struggle with trauma” [1,2], refers to the transformative quality of responding to highly adverse events [3,4], which may be present in addition to the negative sequelae (i.e., posttraumatic stress symptoms) that has been the focus of most of the trauma literature. Although the majority of studies on posttraumatic growth have focused on adults, studies increasingly shows that children can also experience posttraumatic growth [4]. In addition, support was found for similar determinants of growth in adults and children, including stress reactions, event related and demographic characteristics, and social processes [4,5,6]. However, whereas some studies on adults have established different *domains* of posttraumatic growth (e.g., the experience of improved relationships or increased personal strength), studies on children have investigated growth as one broad construct. The current study therefore aims to examine correlates of posttraumatic growth in children, distinguishing between general and domain-specific effects.

### Adversity, stress and posttraumatic growth

Posttraumatic stress and growth have been suggested to co-occur [7,8]. The cognitive and psychological processes in the aftermath of adversity, such as rumination in an attempt to find meaning in the experience, are thought to trigger both posttraumatic stress reactions (i.e., reactions to the traumatic event in the weeks, months or years after the event in term of intrusion, avoidance, arousal etc.) and posttraumatic growth [9]. It is probably not the objective nature of the event, but rather an individual’s subjective stress experience and subsequent stress reactions, which determines whether and how much growth is experienced. Specifically, ‘objective’ trauma severity has previously been found to be only moderately related to subsequent growth [7,10,11]. In contrast, research in children as well as in adults demonstrated substantial positive correlations between *subjective stress experience* and posttraumatic growth [4,5].

### Distinguishing between domains of growth

Posttraumatic growth can manifest itself in various domains, including relationships with others and perceptions of oneself or one’s philosophy of life [2,9,12,13,14]. Consequently, when the Posttraumatic Growth Inventory (PTGI; [12]), was developed, five domains of growth were distinguished: *New possibilities*, *relating to others*, *personal strength*, *spiritual change* and *appreciation of life*. These domains all represent the paradox that “out of loss there is a gain” ([9], p. 6), but allow a further qualification of the experience of growth. Differentiating between domains might be particularly important since overall posttraumatic growth is often a rather subtle experience that co-occurs with more dominant negative and stress-arousing reactions to a traumatic experience (i.e., effects of trauma on stress reaction are often substantially stronger than those on growth experiences; [7,8]). Effects of trauma on growth may be small either because the effects are rather weak, or because the effects are very domain specific and individuals differ substantially with regard to the domain in which they experience growth. Supporting the last, although the majority of studies still lump together the five domains, the possibility that posttraumatic growth experiences differ between individuals and as such, differentially co-vary with various individual characteristics has recently been adopted in adult research [15–18]. For example, in a sample of Australian undergraduate students, Morris and colleagues [17] found that time lag (i.e., time passed since the event) was (negatively) related to the domain *relating to others*, but not to any of the other domains of growth.

### Correlates of posttraumatic growth in children

Whether the domain specific findings in adults translate to school-age children is largely unknown. However, several studies have examined child characteristics in relation to overall child posttraumatic growth. In a systematic review, Myerson and colleagues [4] provided broad support for associations between stress reactions and growth, while the evidence for associations with event, demographic and social characteristics was often mixed. For example, whereas one study on children aged 13–16 years reported more growth in girls than in boys (e.g., [11]), other studies on posttraumatic growth in children described that gender differences had not (yet) been discovered in children ([4,7] for a meta-analysis and an empirical study using the current sample), in contrast to the consistent gender differences found in adults, with women reporting more posttraumatic growth than men [19]. Also, children who identified with a religion as well as children who reported more support from peers, showed sometimes more posttraumatic growth than children who reported no identification with religion or less peer support [11,20,21]. To date, only studies by Laufer and by Wolchik and colleagues have taken into account the specific domains of posttraumatic growth in children aged respectively 13–16 years and 8–16 years [11,22,23]. In their Jewish Israeli sample, Laufer and colleagues found that religious children showed higher levels of *spiritual change* and *appreciation of life* than traditional (i.e., modestly religious) children, who reported higher scores than secular youth. Religious and traditional children scored higher than secular children on the domain *relating to others*, and traditional children scored higher than religious and secular children on the domains *personal strength* and *new possibilities*. Wolchik and colleagues reported a positive association between age and *appreciation of life*, and negative associations between time elapsed since the event and *relating to others* as well as *appreciation of life* [11,22,23]. Seeking adult (but not peer) support was significantly related to all domains of growth except *spiritual change* a few years later. In summary, only two studies so far have examined the domain-specific correlates of child posttraumatic growth, and both these studies used an older child sample. It remains speculative whether the findings from these studies on older children (i.e., adolescents) generalize to younger children.

First, since no studies have assessed domains of growth in younger children, it is unclear whether the specific domains exist at all in younger children. The lack of focus on different domains in child samples may partly be explained by the measurement of posttraumatic growth in children. Whereas the studies by Laufer and colleagues and by Wolchik and colleagues used the Posttraumatic Growth Inventory for (PTGI) to assess posttraumatic growth in older child samples, the majority of studies on posttraumatic growth in younger children have used the child-version of the PTGI, the Posttraumatic Growth Inventory for Children–Revised (PTGI-C-R; [4,24]). It uses wordings and response metrics better suited for children. However, it consists of only ten items, which has led researchers to report on sum scores. Nevertheless, the PTGI-C-R has been designed to parallel the five domains of the PTGI. Although the number of items for each domain is very limited, the inventory may allow an exploration of domain specific associations between various child characteristics and posttraumatic growth in children. As such, before examining such domain specific correlates, the current study examined whether the PTGI indeed allows differentiating between the five domains of growth. If these domains exist in children, this justifies the use of the specific domains in the current study and additionally informs us of the potential need for the development of a more sophisticated instrument.

However, even if the domains are appropriate in younger child samples, it might be that not all previous findings from on older child and adult samples generalize to younger children: Whereas some of the previous findings may translate to young children, other findings may not so easily generalize to a young sample. For example, given the fast and substantial social-developmental changes typical for the years between childhood and adulthood, we are unsure whether for example the findings of Wolchik regarding a link between adult, but not peer, support and posttraumatic growth translates to our younger child sample [23]. As such, exploring the domain specific correlates of children’s posttraumatic growth can help increase our understanding of the positive psychological processes related to children’s well-being after exposure to trauma.

### Current study

The purpose of the current study was to explore correlates of the five domains of posttraumatic growth in a large sample of children exposed to adverse events. We investigated how child demographics, time lag since the event, stress reactions, peer support, and religiosity were related to posttraumatic growth in children. We aimed to differentiate between *general effects* (factors that contribute to all domains of growth) and *domain-specific effects* (factors that contribute to one or more but not all domains of growth). Specifically, based on previous research on posttraumatic growth in children, it was hypothesized that the six child characteristics (child demographics, time lag since the event, stress reactions, peer support, and religiosity) were all related to overall posttraumatic growth. Associations with the specific domains of growth were more exploratory given the lack of literature on domains of growth in children.

To the best of our knowledge, this is the first study disentangling the associations between a range of child characteristics and different domains of growth. Differentiating between domains in children and studying its correlates is important for two reasons: First, since the five domains have been suggested to reflect somewhat disparate psychological processes in adults [14], they may increase our general understanding of the long-term outcomes of trauma exposure in children as well [13]. Second, since children may differ with regard to the specific domains in which growth is experienced, studying general as well as domain specific correlates of growth could contribute to the development of person-centred, tailored interventions with a focus on positive psychological processes.

## Methods

### Participants and protocol

Thirty-six randomly selected schools in Utrecht, a province in the middle of the Netherlands, participated in the study, with 3787 potential respondents (aged 8–12 years) in the last four grades of primary school. A total of 1770 children, whose parents signed informed consent (via an opting-in procedure) and who were present on the day of the data collection, filled out the questionnaires. The study protocol, including the consent procedure, was approved by the Medical Ethics Committee of the University Medical Centre Utrecht. Parents/guardians provided written informed consent for the children. Children who attended school on the day of data collection and whose parents had provided consent, were free to participate or not. All participated and filled out questionnaires in quiet classroom setting (see Alisic and colleagues for details on the procedures; [7]). For the current study we selected those children who reported an adverse event (n = 1290). The mean age of the children was 10.32 years (SD = 1.18). Slightly more girls (52.2%) than boys (47.8%) were included in the current study, but this did not differ substantially from the proportion of girls in the larger sample (50.2%).

### Measures

#### Adverse events

The children were asked whether or not they were exposed to a stressful or traumatic event. Eleven adverse events were listed (i.e., disaster, accident, war, domestic violence (self or other), community violence (self or other), sexual assault, injury/death loved one, serious medical condition and other adverse event). Subsequently, the children were asked to describe their worst experience ever (this could be either one of the events reported before or another event) and to indicate how long ago it took place. Exposure to an adverse event was considered present when the described event fulfilled the A1 criterion for PTSD of the DSM-IV-TR. Two raters independently decided whether the event fulfilled the criterion or not. In case of disagreement (Cohen κ was .58), a third rater made the final decision. Criterion A2 for PTSD was not examined because of possible recall bias. For the current study we included children exposed to both traumatic and non-traumatic (but seriously upsetting) events and took differences between them into account by including severity of the event as a covariate.

*Posttraumatic growth*. The Revised Posttraumatic Growth Inventory for Children (PTGI-C-R, psychometrics see; [24]) is an adaption of the Posttraumatic Growth Inventory, which is frequently used in adults. For the PTGI-C-R, 10 of the original 21 items have been selected that are well accessible to children. The 10 items have a 4-point Likert scale (ranging from 0 = no change to 3 = a lot of change) and a “don’t know” option. For the Dutch version, a back translation procedure has been carried out. Cronbach α for the total scale in the current study was .85. So far, the PTGI-C-R has been used as one broad scale reflecting overall growth. Similar to the adult version, however, the scale reflects five key domains of growth: *New possibilities*, *relating to others*, *personal strength*, *spiritual change* and *appreciation of life* (two items for each domain). For the current study we focused on the five specific 2-item scales. Since, to our knowledge, the specific scales had not been used before, a confirmatory factor analysis (CFA) was performed in Mplus to examine whether the five domains existed and were appropriate in a child sample. The CFA demonstrated that a 1-factor solution did not fit the data very well (χ^2^ = 552.89, df = 35, p < .001; CFI = .834, TLI = .786; RMSEA = .107). In contrast, although the correlations between the 5 factors where rather high (ranging from *r* = .29 to *r* = .61), model fit was adequate in the 5-factor solution (χ^2^ = 109.44, df = 25, p < .001; CFI = .973, TLI = .951; RMSEA = .051). This suggested that the 5-factor solution not only paralleled, but even exceeded the 1-factor solution and as such, are appropriate to use in the current child sample.

#### Predictors of posttraumatic growth

Six demographic, event, stress, and social characteristics were taken into account: age (continuous), gender (dummy), time elapsed since the event (dummy; 0–6 months vs. more than 6 months, [7]), stress reactions (continuous), peer support (continuous), and religiosity (dummy). Stress reactions were measured using the total score on the Dutch version of the Children’s Responses to Trauma Inventory (for psychometrics see: [25]). The CRTI consists of 34 items indicating to what extent a reaction to a traumatic event (i.e., in terms of intrusion, avoidance, arousal etc.) was present during the past 7 days (scores ranged from 1 to 5; α = .92). Peer social support was assessed using the “peers and social support” dimension of the KIDSCREEN-27 (for psychometrics see: [26]). This scale consists of 4 items (α = .75). Questions concern the last 7 days and answers are given on a 5-point Likert scale. Religiosity was assessed using a single item indicating whether the child reported to be a) non-religious, b) Christian, c) Muslim or d) religious but not Christian or Muslim (with a comment box to write down their religion). For this study we recoded the item into a dummy variable indicating whether or not the child identified with a religion.

### Statistical analyses

Missing data totalled less than 3%. Respondents were removed if they had more than 60% missing data on the PTGI-C-R, KIDSCREEN or CRTI (n = 480). Missing values in other cases were imputed using latent class modelling and two-way imputation for separate scales [7].

Multiple regression analyses were conducted using SPSS 20. In the multiple regression analyses, *new possibilities*, *relating to others*, *personal strength*, *spiritual change* and *appreciation of life* were included as dependent variables, and time lag, gender and religiosity (fixed factors) and stress reactions, age, peer social support (covariates) were included as independent variables. The analysis was controlled for the severity of the event children were exposed to (traumatic vs. non-traumatic but seriously upsetting) by including this variable as an additional covariate. This, however, did not change any of the current findings.

## Results

### Descriptive statistics

The following events were reported by the children as their worst experience: disaster (n = 52), accident (n = 256), domestic violence directed at self (n = 17), domestic violence directed at a family member (n = 14), community violence directed at self (n = 50), community violence directed at other (n = 32), sexual assault (n = 7), injury/death of a loved one (n = 523), serious medical condition (n = 94), burglary/theft (n = 6), divorce/parental problems (n = 53), bullying without physical violence (n = 34), death of a pet (n = 96) and other (n = 56; e.g. witnessing a suicide attempt). Descriptive statistics of the predictor and outcome variables are reported in Table 1.

**Table 1:** Descriptive statistics (N = 1290).

		N (%)	Min	Max	Mean	SD
**PTG**	Total		7,4	30	11.73	7.47
	New possibilities		0	6	2.28	1.97
	Relating to others		0	6	2.56	1.92
	Personal strength		0	6	2.79	1.94
	Spiritual change		0	6	1.66	2.08
	Appreciation of life		0	6	2.45	1.92
**Correlates**	Stress reactions		34	150	66.9	22.03
	Age		7.4	13.7	10.32	1.18
	Peer social support		4	20	17.36	2.46
	Time lag (> 6 months)	965 (74.8)				
	Gender (girls)	673 (52.2)				
	Religiosity (yes)	557 (43.2)				

### Child characteristics and posttraumatic growth

The multiple regression analysis showed that almost all predictors under study were significantly related to posttraumatic growth as indexed by the five domains. Stress reactions were positively related to overall posttraumatic growth (F(5, 1278) = 60.64, *p* < .001). Girls reported more posttraumatic growth than boys (F(5,1278) = 3.42, *p* = .004). Older children reported less growth after their traumatic event than younger children (F(5,1278) = 6.67, *p* < .001). Higher levels of social support from peers were related to more posttraumatic growth (F(5,1278) = 3.93, *p* = .002). Children who identified with a religion reported more posttraumatic growth than children who were not religious (F(5,1278) = 60.56, *p* < .001). Only time lag was not significantly related to overall growth (F(5,1278) = 2.13, *p* = .058).

With regard to the five domains, between subject effects and parameter estimates are reported in Table 2. Stress reactions were positively related to all specific domains of growth. For the domain *relating to others*, this was the only significant predictor. Girls reported more growth in all domains except spiritual change. Children for whom the time lag since the adverse event was larger, reported more growth in the domain *new possibilities* than children who experienced the adverse event more recently. A larger time lag was also related to more growth in the domain *personal strength*: children who experienced more peer support also reported elevated levels of growth. Younger children and children who affiliated with a religion reported more *spiritual change*. Finally, younger children and children who experienced more peer support reported more growth with regard to *appreciation of life*.

**Table 2:** Associations between child characteristics and the domains of posttraumatic growth.

PTG subscale	Predictor	B	SE	t	*p*	*ɳ^2^*
**New possibilities**	Stress reactions	.03	.00	12.40	< .001*	.107
	Time lag	.33	.12	2.79	.005	.006
	Age	-.07	.05	-1.56	.119	.002
	Gender (girls)	-.36	.10	-3.46	< .001*	.009
	Peer support	.01	.02	.26	.793	.000
	Religiosity	-.00	.10	-.02	.983	.000
**Relating to others**	Stress reactions	.04	.00	14.76	< .001*	.145
	Time lag	.11	.11	.92	.357	.001
	Age	-.02	.04	-.55	.585	.000
	Gender (girls)	-.13	.10	-1.34	.181	.001
	Peer support	.04	.02	1.83	.068	.003
	Religiosity	.14	.10	1.38	.167	.001
**Personal strength**	Stress reactions	.03	.00	11.83	< .001*	.099
	Time lag	.31	.12	2.65	.008	.005
	Age	-.02	.04	-.47	.642	.000
	Gender (girls)	-.36	.10	-3.51	< .001*	.010
	Peer support	.04	.02	1.92	.055	.003
	Religiosity	-.03	.10	-.31	.757	.000
**Spiritual change**	Stress reactions	.02	.00	9.57	< .001*	.067
	Time lag	.16	.12	1.38	.169	.001
	Age	-.24	.04	-5.36	< .001*	.022
	Gender (girls)	-.19	.10	-1.88	.060	.003
	Peer support	.04	.02	1.98	.058	.003
	Religiosity	1.61	.10	15.69	< .001*	.161
**Appreciation of life**	Stress reactions	.03	.00	13.75	< .001*	.129
	Time lag	.17	.12	1.51	.131	.002
	Age	-.11	.04	-2.50	.013	.005
	Gender (girls)	-.27	.10	-2.70	.007	.006
	Peer support	.08	.02	3.64	< .001*	.010
	Religiosity	-.18	.10	-1.75	.081	.002

Note. *effects that remained significant after Bonferroni corrections.

### Posthoc analyses

Given the large number of comparisons we examined posthoc which effects were most robust. This was done using the highly conservative Bonferroni correction (*p*/number of hypotheses tested). All significant effects of stress reactions, social support and spirituality held after the correction. For age, the significant effect on *spiritual change* held, whereas the effect on *appreciation of life* did not remain significant. For gender, the significant effects on *personal strength* and *new possibilities* held, whereas the effect on *appreciation of life* did not remain significant. Finally, the effects of time lag on *new possibilities* and *personal strength* did not remain significant.

## Discussion

Little is known about the five domains of posttraumatic growth and their determinants in children. In a large sample of 8–12 year old adversity-exposed children in the Dutch general population, we examined associations of child age, gender, time passed since the event, stress reactions, peer support, and religiosity with general and domain-specific posttraumatic growth. This study shows that both general and domain-specific characteristics can be distinguished. That is, whereas some predictors are related to (almost) all domains, others are related to one or two domains of posttraumatic growth only. We will discuss the different findings in turn, as well as their implications for both research and clinical practice.

### General predictors of growth

#### Stress reactions

Stronger stress reactions were related to more growth in general and to each of the domains specifically. Moreover, of all child characteristics, stress reactions were most strongly and consistently related to the five domains of growth. This finding might explain the consistently reported association between stress reactions and overall posttraumatic growth in the literature, in contrast to the more contradictory results for other child characteristics [4].

*Gender*. Gender was also related to most domains of growth: girls reported more growth in all domains except for the domain *relating to others*, for which the effect was non-significant but in the same direction. The finding that gender effects were rather consistently found across the domains may be surprising given the mixed results reported by Meyerson and colleagues [4]. It might be that previous studies did not find any effect due to a combination of modest sample sizes and small effects. In the current study the sample size was rather large, enabling to reveal more subtle effects. Moreover, the consistency of the effect across domains suggests that the associations were robust in their generalisability (although all effects were small, and some did not hold after Bonferroni corrections). It may be that gender differences in posttraumatic growth are small during childhood and increase from early adolescence (possibly as a result of girls’ greater vulnerability to rumination about stressful events than males [4,27]). This would be in line with the findings from adult studies, suggesting that perceptions of growth were up to twice as high for women as for men [12]. Alternatively, the higher levels of reported growth in women may be a result of women’s greater vulnerability to ruminate about stressful events than men’s, which in the end, may result in a stronger sense of growth after adverse life experiences [4,27].

### Domain-specific effects of growth

#### Peer support

Children who reported more support by peers experienced slightly more growth with regard to appreciation of life. The findings seem to suggest that peers may help children become aware of their (increased) growth. However, the effect was small. This suggests that even if support by peers enhances growth, it is not a necessary condition to experience growth [4,5,28]. Possibly, support from others, such as siblings and parents, may be of equal (or even larger) importance. Future research examining multiple sources of support might further investigate this.

#### Time lag

With regard to time lag, no significant overall effect was found and domain specific effects were limited to two domains (*new possibilities* and *personal strength*) and very weak. This might be surprising since previous studies did found effects of time lag on posttraumatic growth [29]. It might be that in the current study time lag was too short to have an effect above and beyond the effects of the other child characteristics. This would be in line with Cohen and colleagues [29], who have suggested that certain aspects of growth might not be evident until years after an event (e.g., growth from child abuse [30]). Also, it may not be the time lag as such, but rather the (psycho)therapeutic treatment children receive which contributes to posttraumatic growth.

#### Age

Older children showed less *appreciation of life* and *spiritual change* (but not other specific domains of growth) than younger children. Previous literature on age as a predictor of posttraumatic growth has produced inconsistent findings. Whereas some studies on overall posttraumatic growth suggested more growth in older than in younger children (e.g.,[20,31,32], other studies have also provided support for a negative association between age and posttraumatic growth [11,21,33], and our previous study [7]). However, besides our previous study [7], all of these studies regarded older samples which makes it difficult to interpret the current findings. Moreover, since childhood is characterised by large developmental changes in a variety of domains (biological, psychological and social) it may not be appropriate to compare the findings from our child sample with those from older child and adult samples. A possible explanation for the inconsistent age effects, however, is that age effects are not only domain-specific but also non-linear: There may be multiple ages during which individuals experience increased posttraumatic growth. However, no longitudinal studies have been performed yet to investigate age effects in detail.

#### Religiosity

Children who were identified as religious reported substantially more *spiritual change* than those who did not [11]. The lack of relations between religiosity and the four other domains suggests that the opportunity of a child to experience *spiritual change*, is context-dependent (i.e., dependent on growing up in a religious family; [24]).

Taken together, our findings provide support for stress reactions and gender as rather general determinants of posttraumatic growth that are consistent (although often small in magnitude) across the different domains. Peer support, time passed since the event, age, and religiosity were all related to only one or two domains of posttraumatic growth. Surprisingly, except for stress reactions none of the child characteristics were related to the domain *relating to others*. Even gender, which was related to growth in all other domains, was not significantly related to an increased sense of compassion, intimacy, or closeness with others. Given that mean levels of *relating to others* were similar to mean levels of most other domains, it appears that children with greater stress reactions experience more meaningful interpersonal relationships regardless of demographic or social characteristics.

### Limitations

Although conducted within a large random sample in the general population, this study should be seen as only a first step in understanding the (determinants of) the domains of posttraumatic growth in children. First, because of the cross-sectional nature of this study, we were not able to distinguish correlations (i.e., concurrent associations between individual characteristic and posttraumatic growth) from causal effects (i.e., the effect of individual characteristic on the amount of growth experienced). This might particularly be a problem with regard to the associations between religiosity, peer support and stress reactions, and growth, where associations might be reciprocal or even reverse. Clearly, for time lag, age and gender this is less of a problem since growth is unlikely to prospectively predict these characteristics. Nonetheless, future studies including multiple waves could disentangle the nature of the associations in more detail. Additionally, it would be interesting to examine how posttraumatic growth develops over time, and whether and how such growth experiences differ from overall growth experienced in people not exposed to traumatic experiences. However, this was both conceptually and methodologically beyond the scope of the current study.

Second, the strength of the effects needs consideration. Only the associations between stress reactions and posttraumatic growth (all domains) and between religiosity and *spiritual change* were substantial in effect size. All other associations were very small. Moreover, four out of the in total 14 significant associations did not hold after the Bonferroni corrections. This may explain 1) why previous empirical studies, including other variables, sometimes found different associations [11,22,23], and 2) the non-consistent findings reported in reviews and meta-analyses both in children and adults [4–6] and shows the need for more in-depth research to understand the important drivers for growth.

Third, in the current study we were unable to take into account treatment the children might have received. Although treatment effects were beyond the scope of this study, treatment may affect the link between trauma exposure and posttraumatic growth. Future studies, preferably using longitudinal designs, might take into account the role of treatment. Fourth, given the already large number of comparisons in the current study, we were unable to test for possible interaction effects, such as gender X age effects, on posttraumatic growth. Examining inter-individual differences in the various links between child characteristics and posttraumatic growth might be a valuable future direction.

Finally, although the validity of the division of posttraumatic growth in five domains in adults has been extensively studied [9] and could be justified in the current study based on the confirmatory factor analysis, this needs further investigation in children. Future studies (including qualitative studies) should examine the concept of posttraumatic growth during childhood in more detail and develop more elaborate questionnaires to fully capture the domains of growth in children. Careful assessment of children’s posttraumatic growth in various domains may be important for clinical practice to be able to both integrate the sense of growth children may experience and recognize vulnerabilities that persist in the aftermath of trauma exposure. For example, knowledge on children’s sense of growth in certain domains (e.g., *personal strength*) may be used to increase coping skills by working from a strength-based approach.

### Conclusion

To our knowledge this is the first study to examine how demographics, event characteristics, stress reactions, and social characteristics are related to the five domains of posttraumatic growth in primary school age children. Although the analyses revealed that all variables were significantly related to posttraumatic growth, there was a distinction between general and domain-specific effects: some characteristics (e.g., stress reactions) were related to all domains of posttraumatic growth, while others were only related to certain domains of growth (e.g., religiosity was only related to *spiritual change*). Whereas most effects were rather small in magnitude, the predictive effects of stress reactions and religiosity were substantial. The present study adds to the literature in various ways. First, by focussing on the positive psychological processes that may co-occur with the traditionally studied negative effects, the current study widens our view regarding the long-term consequences of childhood trauma. Second, the current study is the first examining the appropriateness of differentiating between specific domains of growth in a childhood sample. Finally, understanding general as well as domains specific correlates of childhood posttraumatic growth, even when the effects are small, allows a more person-centred approach and may therefore contribute to the development of tailored interventions.
